# Polystyrene Nanoplastics as Carriers of Metals. Interactions of Polystyrene Nanoparticles with Silver Nanoparticles and Silver Nitrate, and Their Effects on Human Intestinal Caco-2 Cells

**DOI:** 10.3390/biom11060859

**Published:** 2021-06-09

**Authors:** Josefa Domenech, Constanza Cortés, Lourdes Vela, Ricard Marcos, Alba Hernández

**Affiliations:** 1 Group of Mutagenesis, Department of Genetics and Microbiology, Faculty of Biosciences, Universitat Autònoma de Barcelona, Cerdanyola del Vallès, 08193 Barcelona, Spain; josefa.domenech@uab.es (J.D.); constanza.cortes@uab.es (C.C.); lourdes.vela@e-campus.uab.cat (L.V.); 2 Facultad de Ciencias de la Salud Eugenio Espejo, Universidad UTE, Quito 170527, Ecuador

**Keywords:** nanopolystyrene, silver, complexes silver-nanopolystyrene, cell uptake, cell fate

## Abstract

Environmental plastic wastes are continuously degraded to their micro and nanoforms. Since in the environment they coexist with other pollutants, it has been suggested that they could act as vectors transporting different toxic trace elements, such as metals. To confirm this, we have assessed the potential interactions between nanopolystyrene, as a model of nanoplastic debris, and silver compounds (silver nanoparticles and silver nitrate), as models of metal contaminant. Using TEM-EDX methodological approaches, we have been able to demonstrate metal sorption by nanopolystyrene. Furthermore, using Caco-2 cells and confocal microscopy, we have observed the co-localization of nanopolystyrene/nanosilver in different cellular compartments, including the cell nucleus. Although the internalization of these complexes showed no exacerbated cytotoxic effects, compared to the effects of each compound alone, the silver/nanopolystyrene complexes modulate the cell’s uptake of silver and slightly modify some harmful cellular effects of silver, such as the ability to induce genotoxic and oxidative DNA damage.

## 1. Introduction

Plastic demand is continuously growing in different areas such as construction, packaging, automotive, agriculture, mechanical engineering, medical applications, and electronics, among many other fields. Interestingly, about 40% of the produced plastic is dedicated to packaging, constituting the so-called single-use plastics, with a large proportion of these ending up in the environment as waste. Data from 2018 indicate that 9.4 million tons of plastic waste were collected in Europe to be recycled, but 359 million tons of different plastic materials were produced in the world during that year [1]. This means that most of the used plastics do not enter the recycling chain, ending as waste in the environment, thus generating a relevant environmental challenge.

Although the environmental pollution by plastics is an observable fact, this is only the tip of the iceberg. Plastic waste is under a continuous degradation process leading to the formation of micro and nanoplastics (MNPLs) [2] by different environmental conditions or even due to our daily life actions [3]. MPLs encompass particles below 5 mm, while those with sizes ranging from 1 to 100 nm are considered NPLs [4]. MNPLs are ubiquitously present in all ecosystems, including soils, air, and marine and freshwater [5,6]. In this way, their accumulation in different tissues of marine organisms, drinking water, or other daily consumer products has been reported to have a considerable impact on the food web [7,8].

Although humans’ exposure to MNPLs can be through airborne inhalation or dermal exposure, ingestion due to food chain contamination is considered the main entry route of MNPLs [9]. The biological response to MNPLs exposure has been studied in different models such as birds and, especially, in aquatic animals [10,11,12,13], but few published studies focus on analyzing the effects of MNPLs in human models [13]. Since there are no biomonitoring studies in human exposed populations, most of the existing data come from in vitro studies using different human cell models such as fibroblasts, blood cells, lung cells, glioblastoma-derived cells, or intestinal cells. Although different responses have been reported in such studies, MNPLs are generally characterized by a prominent cellular uptake and low or non-existent toxic effects [14,15,16]. This has prompted several authors to propose that MNPLs real toxicological risk could be associated with their potential role as carriers of different environmental pollutants.

Several studies have demonstrated that MNPLs can act as vectors transporting several toxic trace elements, such as organic contaminants or trace metals [17,18,19]. In particular, the interaction between plastic and metals has recently attracted significant attention, emphasizing the ability of MNPLs to adsorb and transport these environmental pollutants [20,21,22,23,24]. Similar to what has been described for MNPLs, metal pollution is widespread over the soil, water, and air worldwide [25,26,27]. Thus, MNPLs and heavy metal particles coalesce in the same environments, fostering the formation of MNPLs/metal complexes, which are spread over terrestrial, aquatic, and aerial environments coming into contact with the food webs. Studies regarding the effects of MNPLs and their associated pollutants or additives on living beings are very scarce, only a few pieces of literature can be found which analyze the impact of these complexes on terrestrial invertebrates, marine organisms, or microorganisms [28,29,30,31,32]. Accordingly, the study of the effects of MNPLs and their adsorbed metals urgently demands a hazard assessment.

Because ingestion is one of the major exposure routes to MNPLs and their adsorbed contaminants, the use of human intestinal cells as an in vitro model target seems appropriate. Caco-2 cells, a human colorectal adenocarcinoma-derived cell line, have been widely used as an in vitro model of the human intestinal epithelium to analyze different toxicological endpoints when exposing them to several nanomaterials, including metals and MNPLs [14,33,34,35]. Accordingly, we have analyzed the ability of MNPLs to act as carriers of metals, and, besides, we have used Caco-2 cells to assess the cellular responses to metal/MNPLs complexes. With this aim, we have determined the interaction between polystyrene nanoparticles (PSNPs) and silver nanoparticles and we have evaluated the different toxic and genotoxic endpoints after the exposure of the Caco-2 cells to the PSNPs interacting with silver nanoparticles, or silver nitrate. Besides, we have also determined whether PSNPs modulate the uptake or the toxicological potential of the adsorbed silver materials on Caco-2 cells. The use of two forms of silver, nanoparticles and the ionic surrogate silver nitrate, allows us to determine the relevance of the silver ionization state in the loading capacity of PSNPs. Finally, the use of fluorophore-conjugated PSNPs (in addition to the pristine particles) enables us to track the intracellular fate of PSNPs (or their complexes) by detecting their fluorescence in our cell model.

## 2. Materials and Methods

### 2.1. Dispersion and Characterization of Silver Materials and Polystyrene Nanoparticles

The nanoparticles used for the different assays were characterized by transmission electron microscopy (TEM) and Z-sizer. Pristine PSNPs (PSNPs, PP-008-10) and fluorescent yellow PSNPs (y-PSNPs, FP-00552-2) were obtained from Spherotech (Chicago, IL, USA). The PSNPs (0.05 to 0.1 μm, according to the manufacturer) were used for all the experiments performed except for the visualization studies of the nanoparticles’ internalization, where y-PSNPs (0.04 to 0.09 μm, according to the manufacturer) were tested. The commercial dispersions were diluted in distilled water to 100 μg/mL and carbon-coated TEM grids were dipped into the samples. TEM visualization was carried out on a JEOL JEM-1400 instrument (JEOL LTD, Tokyo, Japan). Additionally, Image J software supplemented with the Fiji extension was used to measure 100 randomly selected PSNPs to determine their mean size. 100 μg/mL dispersions were further characterized in a Malvern Zetasizer Nano ZS zen3600 device (Malvern, UK) to analyze the hydrodynamic size and the Z-potential parameters using dynamic light scattering (DLS) and laser Doppler velocimetry (LDV) methodologies. All the parameters for each sample were measured three times. AgNPs (SEPE5-25M) were acquired from nanoComposix (Prague, Czech Republic). Commercial AgNPs were firstly diluted in 0.05% bovine serum albumin (BSA) and 100% ethanol to a final concentration of 2.56 mg/mL and then dispersed by ultrasonication at 10% amplitude for 16 min in a cold-water bath. The obtained dispersion was diluted to 100 μg/mL for TEM and Z-sizer characterization, following the guidelines described above. AgNO_3_ (ref. 209139) used in this study was obtained from Sigma-Aldrich (Germany) as a salt. AgNO_3_ was diluted and dispersed using the same procedure followed with the AgNPs to avoid differences between the treatments due to the dispersion procedure. During this process, the salt is diluted and the Ag^+^ ions are released. The obtained AgNPs or AgNO_3_ dispersions were used as a stock solution for all the experimental procedures.

### 2.2. Silver/PSNPs Interactions in Dispersion Media

To analyze the potential interactions between silver and PSNPs, after AgNPs/PSNPs, or AgNO_3_/PSNPs treatments the samples were visualized by TEM. For this purpose, AgNPs or AgNO_3_ were incubated with PSNPs in a distilled water dilution at a final concentration of 5 µg/mL AgNPs or AgNO_3_, and 10 or 100 µg/mL PSNPs for 3 h at room temperature. Then, carbon-coated TEM grids were dipped into the samples and visualized on a JEOL JEM-1400 instrument (JEOL LTD, Tokyo, Japan). To demonstrate that silver materials were on the polystyrene surface, both AgNPs/PSNPs and AgNO_3_/PSNPs samples were analyzed by transmission electron microscopy coupled with energy-dispersive X-ray spectroscopy (TEM-EDX) with a TEM JEOL-2011 (200 kV) instrument (JEOL LTD, Tokyo, Japan) combined with an INCA detector (Oxford Instruments, United Kingdom).

### 2.3. Cell Culture and Reagents

The human colorectal adenocarcinoma-derived cell line Caco-2 was originally kindly provided by Dr. Isabella de Angelis (Instituto Superiore di Sanità, Italy). Caco-2 undifferentiated cells (passages 30–45) were maintained in Dulbecco’s modified Eagle’s high glucose medium without sodium pyruvate (DMEM) (Biowest, France) supplemented with 2.5 µg/mL Plasmocin™ (Invivo Gen, San Diego, CA), 1% non-essential amino acids (Biowest, France), and 10% fetal bovine serum (FBS) (Biowest, France) at 37 °C in a humidified atmosphere of 5% CO_2_ and 95% air. Caco-2 cells were subcultured twice a week to maintain a maximum of 80% confluence. 1% trypsin in PBS 1 X was used to detach the cells, and 2% FBS in PBS 1X was used to inactivate the trypsin.

### 2.4. Silver and Nanopolystyrene Treatments

Undifferentiated Caco-2 cells were exposed to different concentrations of AgNPs, AgNO_3_, PSNPs, y-PSNPs, AgNPs/PSNPs, AgNO_3_/PSNPs, AgNPs/y-PSNPs, and AgNO_3_/y-PSNPs to study the induced effects. For this purpose, 150,000 Caco-2 cells were seeded in 12-well plates and grown for 24 h, allowing the cells to attach to the surface of the well. Then, the culture medium was removed and cells were exposed to 1 mL of the corresponding treatment for 24 h. Different treatments were achieved by diluting the previously dispersed nanoparticles in supplemented DMEM. In the case of co-treatments, silver materials and PSNPs or y-PSNPs were incubated together for 15 min before adding the treatment to the cell culture. All the experiments in which co-treatments were added, were performed with 0, 0.1, 0.5, 1 and 5 µg/mL AgNPs or AgNO_3_, combined with 0, 10, and 100 µg/mL PSNPs or y-PSNPs, unless stated otherwise.

### 2.5. Cell Viability Assay

The Beckman Coulter Counter Z2 (Indianapolis, USA) was used to assess the cell viability when Caco-2 cells were exposed to PSNPs, AgNPs, and AgNO_3_. For this purpose, undifferentiated cells were exposed to 0, 25, 50, 100, 125, 150, 175, and 200 μg/mL PSNPs, or to 0, 0.5, 1, 5, 15, 25, and 50 µg/mL AgNPs or AgNO_3_. These values were used to decide the concentrations to be used in the co-treatments. Later, a cell viability study was carried out to assess the cytotoxicity of the co-treatments on the undifferentiated Caco-2 cells. Briefly, previously-seeded undifferentiated cells were exposed to 0, 0.1, 0.5, 1, and 5 µg/mL AgNPs or AgNO_3_, combined with 0, 10, and 100 µg/mL PSNPs, for 24 h. In all the experiments, cells were washed twice with PBS 1X and detached with trypsin 1% for 5 min at 37 °C after the exposure time. Then, cells were resuspended in 2% FBS in PBS 1X and diluted 1:100 in ISOTON^®^ and the number of cells/mL was counted. Each one of the experiments was performed thrice with triplicate samples.

### 2.6. AgNPs and y-PSNPs Intracellular Co-Localization

To analyze if the observed AgNPs and PSNPs interactions remain during the cell treatment and to confirm the internalization of AgNPs/PSNPs complexes, the co-localization of AgNPs and y-PSNPs inside the Caco-2 cells was visualized by using confocal microscopy. For this purpose, undifferentiated Caco-2 cells were seeded 24 h before the experiment in Glass Bottom Microwell Dishes (MatTek, Ashland, MA, USA) and later exposed to 0, 0.5, or 5 µg/mL AgNPs combined with 0, 10, and 100 µg/mL y-PSNPs. After the exposure, samples were washed with fresh DMEM, and nuclei were stained for 15 min at room temperature with 1:500 Hoechst 33342 (ThermoFisher Scientific, Carlsbad, CA USA), while cell membranes were dyed with 1:500 Cellmask™ Deep Red plasma (ThermoFisher Scientific, Carldsbad, CA, USA). The y-PSNPs were visualized thanks to their fluorophore, and AgNPs were detected thanks to their reflective properties. Two different experiments were carried out, and two randomly selected fields per sample were visualized with a Leica TCS SP5 confocal microscope. Imaris 9.5 software was used for image processing.

### 2.7. Quantification of Silver Uptake by ICP-MS

The inductively coupled plasma mass spectrometry (ICP-MS) analysis was performed to determine the amount of silver internalized by the Caco-2 cells. After the exposure to 0.5, or 5/10, or 100 µg/mL AgNPs/PSNPs or AgNO_3_/PSNPs for 24 h, cells were washed thrice with PBS 1X, and 1% trypsin was used to detach them at 37 °C for 5 min. Subsequently, cells were recovered in glass tubes and centrifuged at 200 rcf for 8 min. The supernatant was discarded and the obtained pellet of cells was frozen at −20 °C and stored until the following steps. Samples were digested on a heat block in concentrated HNO_3_ (Merck, supra-pure) at 105 °C for 30 min, and the amount of silver in each sample was determined using an ICP-MS 7500ce device (Agilent Technologies, Santa Clara, CA, USA). The experiments were performed thrice with duplicate samples.

### 2.8. Production of Intracellular ROS

Intracellular ROS production was determined using the dihydroethidium (DHE) (Merck Group, Darmstadt, Germany) assay and measured by flow cytometry. For this purpose, undifferentiated Caco-2 cells were exposed to AgNPs/PSNPs or AgNO_3_/PSNPs for 24 h. Exposure to 100 µg/mL cigarette smoke condensate (CSC) (Murty Pharmaceuticals, USA) for 40 min was used as a positive control. Cells were then washed twice with PBS 1X, trypsinized, and recovered in FACS tubes to be centrifuged at 200 rcf for 5 min. Caco-2 cells were resuspended in 10 µM DHE in PBS 1X to get a final concentration of 1 × 10^6^ cells/mL, and the cells’ suspensions were incubated at 37 °C for 30 min. Next, the samples were kept on ice from here and 5 nm VPR (BD Bioscience, Franklin Lakes, NJ, USA) was added before the analysis, to discriminate dead cells. A BD FACSCanto Flow Cytometer (BD Bioscience, Franklin Lakes, NJ, USA) was used to measure the fluorescence of 10 000 events from the living-cells population using a 488/515–545 nm excitation/emission spectrum. Three independent experiments were carried out with duplicate samples for each condition.

### 2.9. Genotoxic and Oxidative DNA Damage Induction

After exposing undifferentiated Caco-2 cells to AgNPs/PSNPs or AgNO_3_/PSNPs for 24 h, genotoxic and oxidative DNA damage (ODD) were assessed by the alkaline comet assay complemented with the enzyme formamidopyrimidine DNA glycosylase (FPG). For this purpose, after removing the treatments, cells were washed with PBS 1X and detached with trypsin 1% at 37 °C for 5 min. Then, Caco-2 cells were collected, centrifuged at 0.3 rcf for 8 min, and the pellets were resuspended in PBS 1 X to get a final concentration of 1 × 10^6^ cells/mL. Then, 25 µL of the cell suspension was mixed 1:10 with pre-melted 0.75% low melting point agarose at 37 °C. Each sample was dropped on a GelBond film (GBF) (Life Sciences, Lithuania) in triplicates, and cells were lysed with lysis buffer (2.5 M NaCl, 0.1 M Na_2_EDTA, 0.01 M Tris Base, 1% Triton X-100, 1% lauroyl sarcosinate, 10% DMSO, and pH 10) overnight at 4 °C. GBFs were then washed with cold enzyme buffer (0.04 M HEPES, 0.1 M KCl, 0.5 mM EDTA, 0.2 mg/mL BSA), and incubated with pre-heated enzyme buffer, with or without 1:2 500 FPG enzyme, for 30 min at 37 °C. Next, GBFs were washed and incubated with electrophoresis buffer (0.3 M NaOH, 1 mM EDTA) for 35 min at 4 °C, allowing the DNA unwinding to expose the alkali-labile sites. The electrophoresis was run at the constant electric tension of 20 V and 300 mA at 4 °C, and then, GBFs were washed with cold PBS 1 X, and cells were fixed in ethanol 100% for at least 1 h. After being air-dried overnight, GBFs were incubated in 1:1 250 SYBR Gold 10 X (ThermoFisher Scientific, Carldsbad, CA, USA) in TE buffer (10 mM Tris Base, 1 mM EDTA) for cell staining for 20 min at room temperature. For the comets’ analysis, GBFs were mounted on methacrylate support and visualized with an epifluorescence microscope (Olympus BX50) at 20× magnifications. The DNA damage was extrapolated from the percentage of DNA in the comets’ tails using the Komet 5.5 image analysis software (Kinetic Imaging Ltd., Liverpool, UK). For this, one hundred randomly selected comets were quantified per sample, and each sample was analyzed with duplicates in each of the three independent experiments performed. Methyl methanesulfonate (200 μM MMS, Sigma-Aldrich, DarmsstadtGermany) was used as a positive control for general genotoxic damage, while potassium bromate (5 mM KBrO_3_, Sigma-Aldrich, Germany) was used as a positive control for ODD.

### 2.10. Statistical Analysis

All results obtained are the average of three independent experiments with duplicates unless otherwise specified. Data were analyzed with the software GraphPad Prism 5 and different statistical analyses, specified in each result section for each assay, were used depending on the data characteristics. Statistical significance was defined as * *p* ≤ 0.05, ** *p* ≤ 0.01, *** *p* ≤ 0.001. The asterisks above each column refer to the statistical significance regarding the negative (untreated) control, without silver and without plastic. The asterisks above the brackets refer to the statistical significance between the columns indicated by the bracket unless stated otherwise.

## 3. Results

### 3.1. AgNPs and PSNPs Characterization

**Figure 1:**
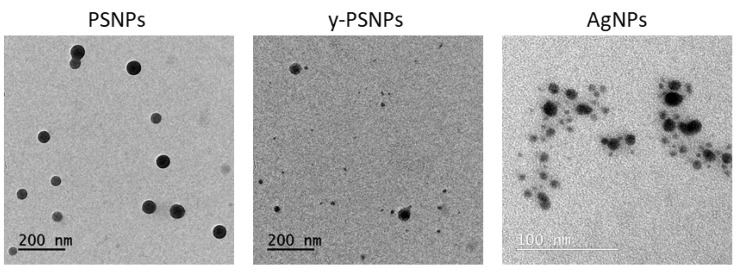
Representative TEM images of PS nanomaterials (PSNPs and y-PSNPs) and AgNPs. Dilutions of 100 μg/mL in distilled water of each material were used for the visualization.

AgNPs and PSNPs were visualized by TEM to determine their morphology and size. Figure 1 shows that PSNPs are composed of electrodense spherical shaped particles of about 40 nm in diameter. Conversely, AgNPs are much smaller spherical shaped particles of about 5 nm in diameter.

Table 1 summarizes the PSNPs, y-PSNPs, and AgNPs median size measured by Image J from TEM images, together with the data obtained using Z-sizer analysis. While PSNPs and y-PSNPs are similar in appearance and size regarding TEM analysis, sizes appeared to be larger when the hydrodynamic radius was measured by DLS. The same happened when AgNPs were analyzed. Besides, the polydispersity index (PdI) values indicate that PSNPs samples are more consistent dispersions than y-PSNPs, as PdI values close to 0 indicate that the sample is monodispersed. Contrarily, PdI values for AgNPs showed a broader particle size range. Even though, results for Z-potential measurements showed that all the dispersions are stable and do not tend to aggregate. These data correlate with what was observed by TEM.

### 3.2. Determination of the Silver/PSNPs Interactions in Solution

**Figure 2:**
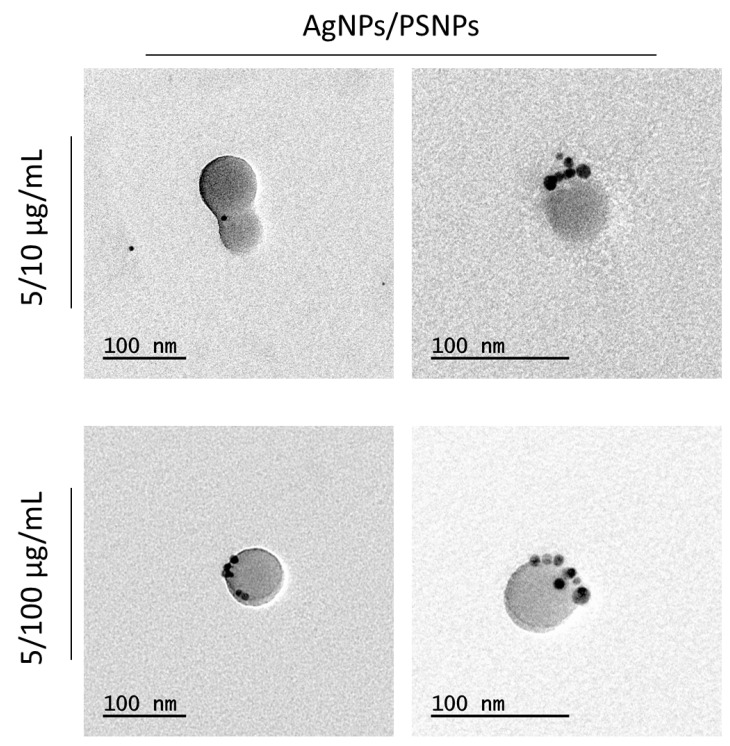
Representative transmission electron microscopy (TEM) images of 5 µg/mL AgNPs mixed with 10 and 100 µg/mL PSNPs. The visualized co-treatments are indicated in the Figure as 5/10 or 5/100 µg/mL.

**Figure 3:**
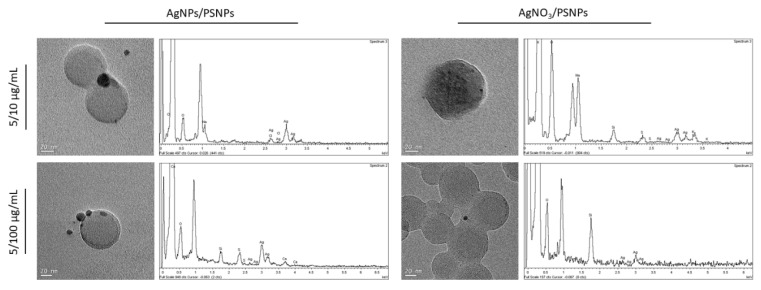
Representative transmission electron microscopy (TEM) images associated with its energy-dispersive X-ray spectroscopy (EDX) spectra showing the chemical elemental characterization. Representative TEM images and its EDX spectra correspond to 5 µg/mL AgNPs or AgNO_3_ mixed with 10 or 100 µg/mL PSNPs (indicated in the Figure as 5/10 or 5/100 µg/mL, respectively).

The interaction between AgNPs and PSNPs particles in dispersion was visualized by TEM, as shown in Figure 2. As observed, electrodense particles were found on the PSNPs’ surface at both polystyrene concentrations used, when testing the 5 µg/mL dose of AgNPs. To demonstrate that the electrodense particles were silver, and to validate the potential co-localization of silver ions and polystyrene particles, elemental silver was tracked by TEM-EDX. The detection of the pick of silver’s electrons transition, as shown in Figure 3 diagrams, demonstrates the co-localization of silver particles or silver ions and PSNPs. For these assays, 5 µg/mL of AgNPs or AgNO_3_ were incubated with 10 and 100 µg/mL PSNPs. This is indicated in Figure 2 and Figure 3 as 5/10 or 5/100 µg/mL.

### 3.3. Cytotoxicity Assessment

**Figure 4:**
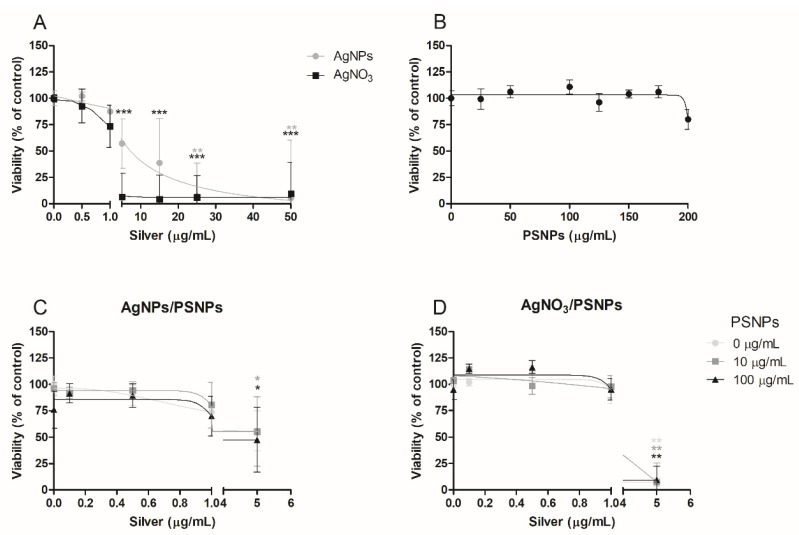
Relative viability of undifferentiated Caco-2 cells after 24 h of exposure to AgNPs or AgNO_3_ (**A**), PSNPs (**B**), AgNPs/PSNPs (**C**), and AgNO_3_/PSNPs (**D**) at concentrations ranging from 0 to 50 µg/mL for silver materials and PSNPs concentrations ranging from 0 to 200 µg/mL. The viability percentages were calculated by averaging the number of cells counted for each condition in three independent experiments. Cell viability is represented as the percentage of counted cells compared to the untreated control ± SEM. Data were analysed by comparing each condition to the untreated control using Student’s t-test. * *p* ≤ 0.05, ** *p* ≤ 0.01, *** *p* ≤ 0.001

A viability assay was performed to assess the cytotoxic concentrations of silver materials, nanopolystyrene, and silver/PSNPs co-treatments on undifferentiated Caco-2 cells for exposures lasting 24 h. As shown in Figure 4A, there is a significant decrease in the cells’ viability after exposure to AgNO_3_ starting from the concentration of 5 µg/mL. However, this decrease in the cell viability is slightly lower when Caco-2 cells were exposed to AgNPs, reaching significant values from 25 µg/mL of treatment. Accordingly, undifferentiated Caco-2 cells are more susceptible to AgNO_3_ than to AgNPs, even though a decrease below 75% of cell viability is observed from the concentration of 5 µg/mL in both treatments. PSNPs show no cytotoxicity over Caco-2 cells (Figure 4B), as cell viability remained unaltered up to 150 µg/mL PSNPs exposures. Furthermore, although cells treated at the highest concentration tested show a decrease in cell viability, it does not reach statistically significant values. On the other hand, the results obtained for the AgNPs/PSNPs or AgNO_3_/PSNPs co-treatments are represented in Figure 4C,D, respectively. In both cases, cell viability is maintained constant at the concentrations 0.1, 0.5 and 1 µg/mL of silver mixed without or with 10 or 100 µg/mL of PSNPs. However, a significant reduction in the cells’ viability is observed at a 5 µg/mL concentration of silver materials, independently of PSNPs, being more pronounced on AgNO_3_/PSNPs treatments. The PSNPs addition does not exert a differential effect in any of the treatments assayed, as cell viability results for Caco-2 cells remain unvaried independently of whether silver materials are mixed or not with PSNPs.

### 3.4. Intracellular Co-Localization of AgNPs/y-PSNPs Complexes

**Figure 5:**
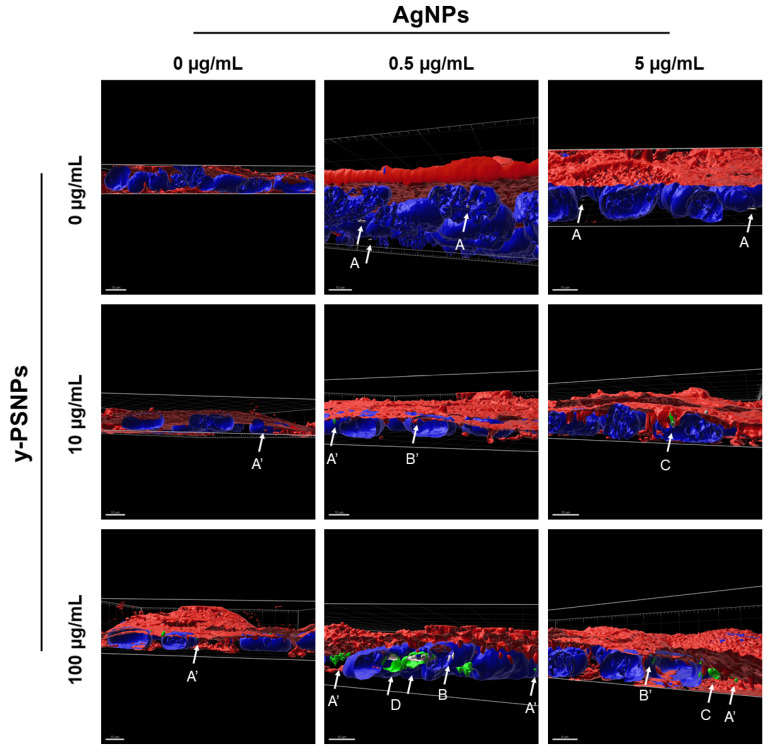
Selection of representative three-dimensional images of the Caco-2 cells exposed to 0, 0.5 or 5 µg/mL AgNPs combined with 0, 10 and 100 µg/mL y-PSNPs for 24 h. Nuclei and cell membranes were stained with Hoechst (blue) and CellMask (red), respectively. y-PSNPs particles are shown in green and AgNPs are shown in grey. Arrows point out: AgNPs and y-PSNPs located inside the cells (indicated as A and A’, respectively); AgNPs and y-PSNPs located inside the nuclei (indicated as B and B’, respectively); AgNPs/y-PSNPs complexes located inside the cells (indicated as C); and AgNPs/y-PSNPs complexes located inside the nuclei (indicated as D). Imaris 9.5 was used to process the images and take the shown snapshots. Two different experiments were carried out and two different randomly chosen fields were analysed.

Confocal microscopy was used to determine the co-localization and cellular location of AgNPs and PSNPs inside the Caco-2 cells after the exposure. On the one hand, silver and PSNPs were found on their own inside the cells in the different conditions analyzed, indicating that there is particle internalization at all the given concentrations for both silver and PSNPs (Figure 5, A and A’ indicators, respectively). Besides, AgNPs and y-PSNPs were able to target/enter the nuclei at the different conditions (Figure 5, B and B’ indicators, respectively). The complexes AgNPs/y-PSNPs were also found inside the cells at the conditions 0.5 + 100, 5 + 10 and 5 + 100 µg/mL AgNPs/y-PSNPs (Figure 5, C indicator). Furthermore, AgNPs/y-PSNPs complexes were visualized inside the cell nucleus, as observed in Figure 5, D indicator, at the 0.5 + 100 µg/mL AgNPs/y-PSNPs condition. To summarizing, AgNPs and y-PSNPs can remain associated, forming complexes during their uptake by the Caco-2 cells and even reach the cell nucleus.

### 3.5. Quantification of Silver Uptake

**Figure 6:**
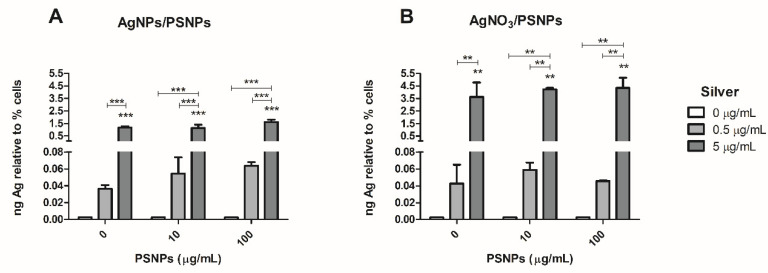
Quantification of the internalized silver by Caco-2 cells after the exposure to 0, 0.5 and 5 µg/mL/0, 10 and 100 µg/mL of AgNPs/PSNPs (**A**) or AgNO_3_/PSNPs (**B**). Data were obtained by averaging three independent experiments performed in duplicates. The duplicates of each experiment were averaged, and the percentage of viable cells was used to normalize the results. ICP-MS device did not detect silver in those samples treated without silver, so these results are arbitrarily represented as 0.0025 ng of silver. Data are represented as mean ± SEM and was analysed by one-way ANOVA with Tukey’s multiple comparison post-hoc test, ** *p* ≤ 0.01, *** *p* ≤ 0.001.

After the exposure of Caco-2 cells to AgNPs/PSNPs or AgNO_3_/PSNPs, silver internalization was quantified by ICP-MS. The amount of silver detected inside Caco-2 cells after the treatment with AgNPs/PSNPs (Figure 6A) showed a significant dose-dependent increase at all the polystyrene concentrations. Also, when comparing the results obtained for each concentration of AgNPs at the different PSNPs doses, a slight tendency to increase silver internalization is observed when PSNPs concentrations are increased. Regarding the AgNO_3_/PSNPs treatments (Figure 6B), a significant AgNO_3_ dose-dependent increase of the internalized silver can be observed at all the polystyrene concentrations, reaching significant values at all the PSNPs concentrations. This increase is much more pronounced than the one observed for AgNPs/PSNPs treatments. Again, a slightly increasing trend is shown in the amount of internalized silver as the polystyrene dose increases when cells are exposed to 5 µg/mL AgNO_3_.

### 3.6. Production of Intracellular ROS

**Figure 7:**
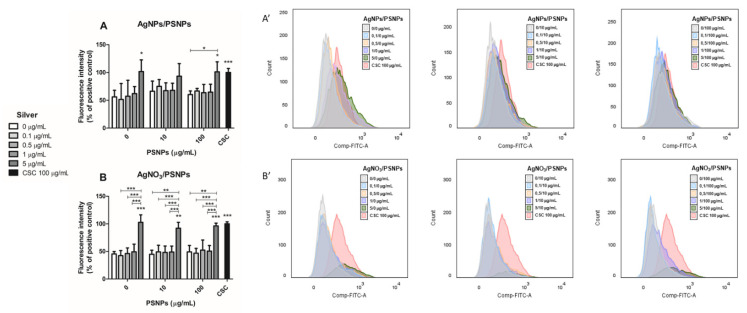
Intracellular ROS production analysis in undifferentiated Caco-2 cells exposed to AgNPs/PSNPs (**A**) or AgNO_3_/PSNPs (**B**) for 24 h. Exposure to 100 µg/mL CSC was used as the positive control. ROS production was extrapolated from the average of the mean fluorescence intensity from three independent experiments with duplicate samples (10 000 events taken from the living cell population of each sample). Data are represented as the percentage of fluorescence intensity compared to the positive control ± SEM and analysed by one-way ANOVA with the Tukey’s multiple comparison post-hoc test. The histograms showing the fluorescence of the living cell population for each sample treated with AgNPs/PSNPs are represented in (**A’**). The histograms showing the fluorescence of the living cell population for each sample treated with AgNO_3_/PSNPs are represented in (**B’**). * *p* ≤ 0.05, ** *p* ≤ 0.01, *** *p* ≤ 0.001

The DHE assay was used to determine the intracellular ROS production in Caco-2 cells after exposure to the co-treatment AgNPs/PSNPs and AgNO_3_/PSNPs for 24 h. Our results demonstrate that both AgNPs/PSNPs and AgNO_3_/PSNPs co-treatments in Caco-2 cells show similar results independently of the PSNPs concentration (Figure 7A,B, respectively). Despite that, there is a tendency to increase the ROS production in the samples treated with the highest concentration of AgNPs (5 µg/mL), reaching significant values similar to those of the positive control. Similarly, the intracellular ROS production reached statistically significant values at the highest concentration of AgNO_3_ assayed in all the co-treatments with PSNPs that are comparable with the positive control. Data are shown as the percentage of fluorescence intensity regarding positive control in Figure 7A,B. The histograms showing the fluorescence of the living cell population for each sample treated with AgNPs/PSNPs or AgNO_3_/PSNPs are represented in Figure 7A’,B’, respectively. As assessed in the viability assay, the highest concentration of AgNO_3_ exert a cytotoxic effect on Caco-2 cells. So that, the number of cells analysed at the conditions containing 5 µg/mL AgNO_3_ is lower compared to the other conditions. Nevertheless, the remaining cells show fluorescence intensity values similar to those in the positive control.

### 3.7. Determination of Genotoxicity and Oxidative DNA Damage

**Figure 8:**
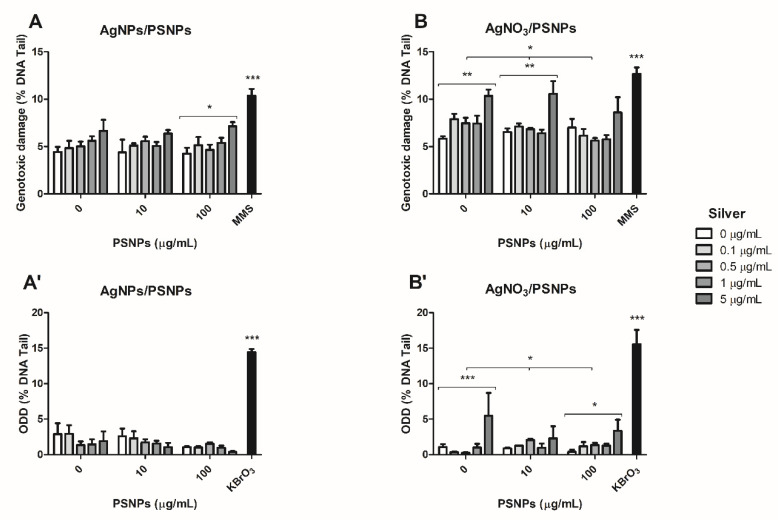
Genotoxic damage detected with the comet assay after 24 h of AgNPs/PSNPs (**A**) or AgNO_3_/PSNPs (**B**) exposures, using 200 µM MMS as a positive control. Oxidative DNA damage (ODD) detected using the FPG enzyme after 24 h of AgNPs/PSNPs (**A**’) or AgNO_3_/PSNPs (**B**’) exposures, using 5 mM KBrO_3_ as a positive control. Three independent experiments were performed with duplicates and 100 randomly selected cells were analyzed by the software. Genotoxic and oxidative DNA damage are extrapolated from the percentage of DNA in the comets’ tail. Data represented is the result of averaging the three experiments. For ODD calculation we subtracted genotoxic damage from general damage for each experiment and averaged the resulting values. Data are represented as mean ± SEM and analysed by one-way ANOVA with linear trend post-hoc test. Brackets indicates the group of samples for which significant linear trend is shown. * *p* ≤ 0.05, ** *p* ≤ 0.01, *** *p* ≤ 0.001

The comet assay, complemented with the FPG enzyme, was performed to quantify the genotoxic and oxidative DNA damage induced by the different exposures. As shown in Figure 8A, there is an increase in the genotoxic damage levels related to the AgNPs concentration. This trend is maintained independently of the PSNPs doses. However, genotoxicity reaches significant linear tendency values only when cells are treated with AgNPs mixed with 100 µg/mL PSNPs (* *p* ≤ 0.05). In the same way, the tendency to increase genotoxic damage at the highest silver concentration is maintained when cells are treated with AgNO_3_/PSNPs (Figure 8B) but, in this case, the treatment exerts a more pronounced genotoxic effect, reaching statistically significant values of linear tendency when the cells are treated with AgNO_3_ alone or mixed with 10 µg/mL PSNPs (** *p* ≤ 0.01, in both cases). The lack of a significant trend in genotoxic effects observed with AgNO_3_/100 µg/mL PSNPs can be explained due to the previously shown cytotoxic effect of the 5/100 µg/mL of AgNO_3_/PSNPs combination. Thus, most DNA-damaged cells did not survive the treatment and, consequently, they are not incorporated in the analysis. In addition, statistically significant values are found in a polystyrene-dependent fashion at 0.5 µg/mL AgNO_3_ (* *p* ≤ 0.05). Regarding the induction of ODD, we did not find oxidative damage in the DNA of Caco-2 cells treated with AgNPs/PSNPs (Figure 8A’). Conversely, the most marked effects of the silver ion are observed again when analyzing the ODD in cells exposed to AgNO_3_/PSNPs, where an AgNO_3_ dose-dependent increasing trend is observed reaching significant values when cells are treated with AgNO_3_ alone (*** *p* ≤ 0.001), or combined with 100 µg/mL PSNPs (* *p* ≤ 0.05). Besides, statistically significant changes are found in a polystyrene-dependent fashion. The exposure of Caco-2 cells to low concentrations (0.5 µg/mL) of AgNO_3_ combined with the different doses of PSNPs shows a significant trend as the PSNPs concentration increases (Figure 8B’).

## 4. Discussion

The ubiquitous presence of plastic waste makes its derivatives micro- and nanoplastics (MNPLs) emergent contaminants. Thus, substantial efforts are required to evaluate their potential health risk for both environmental organisms and humans. Given the lack of human biomonitoring studies and the scarcity of studies in in vivo mammalian models, the use of in vitro studies using mammalian and human cell-line models has become the standard way to evaluate the potential risk posed by nanoplastics [15]. Due to the lack of commercially available nanoplastics, aside from nanopolystyrene, most of the reported studies used this nanoplastic as the standard model. This constitutes an obvious bias in the studies looking for potential risks that environmental nanoplastics pose to humans.

Toxic effects of nanopolystyrene have been reported in cerebral (T98G) and epithelial (HeLa) human cells [36], as well as in human macrophage THP-1 cells [37], and placental trophoblast (BeWo b30) cells [38]. Similarly, negative effects were reported in undifferentiated Caco-2 cells [14,39], as well as in differentiated cells forming part of complex bi-culture (Caco-2/HT29) and tri-culture (Caco-2/HT29/Raji-B) models [16,37,38]. Also, cytotoxicity was reported in human lung epithelial BEAS-2B [40] and in three different human leukocytic cell lines (Raji-B, TK6, and THP-1). In this last study, cellular uptake, reactive oxygen species (ROS) production, and genotoxicity were observed, although with substantial differences among the selected cell lines [15]. These results were confirmed in ex vivo exposures using white cells from the whole blood of different donors. In that study, clear differences between the different cell types were observed for uptake and DNA damage, as well as for the expression of various cytokines [41].

It should be pointed out that most of these studies were conducted using pristine nanopolystyrene samples, which are significantly different from those present in the environment. Thus, evaluating the toxic effects of MNPLs environmental samples is a future challenge. However, as reported by Zhu et al. [5], spherical microplastics, among others, are found in aquatic and terrestrial environments. This can be explained by the use of micro- and nanoplastic beads in the production of cosmetics such as scrub and exfoliating products that end as plastic debris in marine environments [42]. According to such authors, the total production of cosmetic products associated with the use of micro/nanobeads for 2012 in Europe was 6.88x10^8^ L, representing about 4130 tons of micro/nanobeads. As the production levels have been increasing, this gives us an idea of the exposure to micro/nanobeads, and their relevance as environmental plastic wastes.

Independently of their size and composition, environmental nanoplastics can interact with other environmental pollutants due to their hydrophobic nature and their large surface area, which facilitates the adsorption of different contaminants such as polychlorinated biphenyls, polycyclic aromatic hydrocarbons, and heavy metals. Thus, nanoplastics could act as vectors for other more toxic elements present in the environment, potentially affecting their toxicological profiles [43]. Nevertheless, this attractive proposal has been poorly evaluated until now. So, more realistic studies are needed, which take into account the potential sorption ability of nanoplastics for environmental contaminants [44]. In this context, we have evaluated the potential interactions between nanopolystyrene and silver material, as a model of environmental metal pollutants. From this point of view, using commercial pristine particles has the advantage of allowing to set the bases of the methods, technics, and assays suitable to analyse the interactions between environmental nanoplastics and other environmental pollutants with a homogeneous but also, realistic model of nanoplastic particles found in the environment.

It is known that nanomaterials can dissolve in different conditions. This also occurs with AgNPs that can release Ag^+^ ions in solution. We studied in parallel this release, using ICP-MS, observing that about 12% of the used AgNPs (at the concentration of 10 µg/mL) were dissolved at 24 h. Interestingly this release remained stable at least until the 72 h. Nevertheless, this does not affect our study because we included AgNO_3_ as a model of agent-releasing ions. Thus, this comparison permits the discrimination of the effects induced by particles (AgNPs) from those generated by ions (AgNO_3_). Taking into account this small release we did not carry out further solubility studies when AgNPs were combined, assuming no significant changes in the solubility of AgNPs.

Our study clearly shows, by using TEM microscopy, that silver nanoparticles associate with nanopolystyrene. This data reinforces the interest in using silver or other metals in their nanoparticulated form as a way to detect/visualize this type of interaction. Silver nanoparticles, in particular, have attracted the attention of many industries, especially those looking for antiseptic effects, due to their unique properties. These antiseptic characteristics are particularly desirable in food, textile, construction, medicine, cosmetology, pharmacy, and other commodities. Regarding the environmental impact of silver compounds, it should be noted that their estimated market for 2020 is about 600 tonnes, indicating that copious amounts of silver materials are constantly being released into the environment [45].

In addition, TEM images also showed a granulated form of nanopolystyrene when it was incubated with silver nitrate. The confirmation of the sorption of silver nitrate by nanopolystyrene was obtained when TEM was complemented with EDX methodologies, where the obtained spectra showed the chemical elemental characterization of silver. All these results confirm, for the first time, the usefulness of TEM-EDX methodologies to visualize nanoplastic/metal associations.

Currently, there is no general agreement on whether nanoplastics can intensify the toxicity of environmental contaminants although, theoretically, increased adsorption could result in enhanced bioaccumulation [44]. Our results with the two forms of silver do not seem to indicate differences in the toxicity of both silver materials alone or associated with nanopolystyrene exposures. From the two existing studies reporting data on metal/nanoplastic interactions, the study of Davranche et al. [46] showed metal-binding using nanoplastics produced from microplastics collected on the beach, and lead as the metal model. Unfortunately, they did not carry out toxicity studies, although they suggested the interest of assessing the toxic effects of nanopolystyrene-adsorbed metals. On the other hand, in the study of Yan et al. [47], the authors evaluated the toxicity of cadmium, lead, and zinc with nanopolystyrene on marine medaka fish, even though they did not present confirmation of the metal/plastic interaction. The reported data indicated a potential interplay, increasing the toxicity on the intestinal microbiota, as the metal/nanopolystyrene treatment caused a higher pollution load on the gut. Nevertheless, no interaction effects were observed when reproductive disturbances were evaluated by measuring the gonadal development, as well as alterations in the gene expression pattern related to the hypothalamic-pituitary-gonadal (HPG) axis. Consequently, our and these reported data do not seem to support a potential increase in toxicity when metals are bound to nanoplastics.

The interaction between silver compounds and nanopolystyrene, detected in liquid dispersions, was also observed inside the cells. In this scenario, the use of fluorescent polystyrene nanoparticles, together with the reflective properties of silver nanoparticles, permitted their detection inside the cells when confocal microscopy methodology was used. Thanks to the co-localization of both signals, it was confirmed that silver nanoparticles and polystyrene nanoparticles remain associated during their uptake by the Caco-2 cells. Interestingly, these complexes were also observed in the cell nucleus. All these data support the usefulness of confocal methodologies to visualize the fate of these complexes inside the cells. Moreover, it should be remarked that this is a novel approach never used before.

A further question is whether such complexes increase the uptake of metals by the cells. To answer this, we determined the levels of silver inside the cells by ICP-MS when cells were treated with silver materials alone or in combination with nanopolystyrene. The obtained results indicate that at low concentrations, there is a tendency to increase silver materials uptake depending on the concentration of polystyrene nanoparticles, but it does not reach significant values. The lack of differences observed at the highest tested concentration might indicate that a possible saturation threshold has been reached at such concentration. Taking into account that the environmental concentrations of silver materials (or metals) must be low, the presence of nanoplastics could increase the metal uptake and, potentially, increase their toxic effects.

Once the cell internalization of silver nanoparticles/nanopolystyrene complexes was proved, the potential activation of relevant toxicological markers, such as oxidative stress induction and genotoxicity, was evaluated. Oxidative stress induction is a general mechanism associated with nanomaterials exposure [48], and it has been proved that metals, and specifically silver nanoparticles, can induce high levels of intracellular ROS in cultured human cells [49]. Considering the role of oxidative stress induction as a mechanism involved in the harmful effects of micro- and nanoplastic exposures, it has been proposed as the primary driving mechanism affecting exposed marine organisms [50]. Nevertheless, its role in in vitro exposed human cells is not conclusive enough. The results observed in cerebral and epithelial cell lines [36], human dermal fibroblasts, and murine macrophages [39] were not observed in the human colon adenocarcinoma Caco-2 cell line, where no effects were detected in both undifferentiated [14] and differentiated Caco-2 cells [16]. In these last studies, the lack of ROS induction was assessed using two different but complementary assays (DCFH-DA and DHE) and confirmed by detecting no changes in the expression levels of *SOD2*, and *GSTP1* genes, both related to the oxidative stress pathway. This cellular response disparity could be due to the intrinsic characteristics of each type of cell line used. Such differences were recently demonstrated in a study assessing the effect of nanoplastics on different human hematopoietic cell lines, where exposures to nanopolystyrene did not induce intracellular ROS in THP1 cells, although a slight increase was observed in Raji-B cells, and a clear response was shown in TK6 cells [15]. In our case, we have confirmed that nanopolystyrene was unable to produce intracellular ROS in undifferentiated Caco-2 cells. However, it must be pointed out that DHE specifically detects the formation of superoxide anion and hydrogen peroxide species, but not hydroxyl radicals. Thus, we extrapolated intracellular ROS production from the measurement of superoxide anion and hydrogen peroxide. Even though the highest concentration of both silver forms increased ROS production, the association with nanopolystyrene did not modify the effects observed with silver materials alone in a statistically significant way.

Any assessment of the harmful effects of environmental pollutants must include an evaluation of their potential DNA damage, as it has been well demonstrated that DNA lesions induction entails relevant consequences for human health [51]. Nevertheless, and despite the relevance of this biomarker, only a few studies on nanoplastics include genotoxicity among their assessments. Consequently, our study has investigated the potential genotoxic effects associated with the presence of silver materials/nanoplastics complexes. The results, obtained with the comet assay, confirmed that nanopolystyrene alone is not genotoxic in Caco-2 cells, as reported in previous studies in the same cell line [14] where no genotoxic effects were observed either by using the comet assay (measuring mainly DNA breaks) or the micronucleus assay (measuring chromosome breaks and/or chromosome loss). Nevertheless, and as previously indicated for other biomarkers, the genotoxic effects of nanopolystyrene can depend on the used cell line. As observed using three different human hematopoietic cell lines (TK6, Raji-B, and THP), significantly high levels of DNA damage were observed in Raji-B cells, while no effects were detected in neither TK6 nor THP cells [15]. Similarly, different subsets of white peripheral blood cells obtained using cell sorter methodology, namely lymphocytes, monocytes, and polymorphonuclear (PMN) cells, showed different sensitivity when exposed to nanopolystyrene. Also, high levels of DNA breaks (as measured using the comet assay) were detected in PMN cells, while moderate effects were observed for monocytes and no effect for lymphocytes [41]. In our study, we have confirmed the lack of genotoxicity of polystyrene nanoparticles in Caco-2, and the different genotoxic potential of the two evaluated silver compounds, where silver nitrate had higher genotoxicity than silver NPs. Using this biomarker, the effects of silver materials/PSNPs complexes are not apparent when direct genotoxic effects were evaluated, compared to the effects of the silver compounds exposure alone. Nevertheless, it should be noted that a statistically significant genotoxic effect trend of the silver NPs/nanopolystyrene complexes was observed when both compounds were simultaneously evaluated at the highest concentration, as well as when AgNO_3_ was evaluated in combination with the lowest concentration of PSNPs. Interestingly, a significant trend to increase genotoxic damage was found at low concentrations of AgNO_3_ in a polystyrene dose-dependent fashion. This finding must be highlighted since this silver low concentration mimics the environmental conditions. On the other hand, the effects on the oxidative DNA damage of the silver compounds/nanopolystyrene complexes were more evident when the levels of oxidatively damaged DNA bases were evaluated using silver nitrate. In this case, a significant tendency to higher levels of oxidative DNA damage was observed in a polystyrene dose-dependent fashion, when the lowest concentrations of silver nitrate was combined with the different concentrations of nanopolystyrene. Again, this is an environmentally relevant finding. Thus, our results would suggest that at low concentrations of silver, the presence of nanoplastics would increase both the metal uptake and their genotoxic and DNA oxidative damage effects.

It should be stated that although we have not included interaction kinetics studies, we have been able to demonstrate the ability of PSNPs to adsorb environmental pollutants, using silver materials. From this point of view, the objective was completed, since we showed pieces of evidence of the interaction between the PSNPs and AgNPs. In addition, and regarding the stability of the interaction our results indicate that the visualization observed by TEM after 3 h of “incubation” is confirmed 24 h later by using confocal images. In this last case, the complex silver nanoparticles/PSNPs had “travel” into the cells without lost their interaction. This indicates that such interaction remained stable.

An interesting topic refers to the behaviour (aggregation kinetics and stability) of PSNPs in natural waters. This theme has been the subject of studies showing that, although CaCl_2_ concentrations can affect their stability, PSNPs remain more stable than other nanoplastics such as nanopolyethylene. Such stability can be attributed to the surface functionalization (presence of carboxyl functional groups) of PSNPs. Furthermore, the presence of natural organic matter (humic acid) in water improved the stability of PSNPs, primarily due to steric repulsions. Consequently, a significant aqueous transport of PSNPs will be possible in natural surface water [52]. In this way, interactions as those reported in this study can be possible.

## 5. Conclusions

Our study provides methodological evidence showing that nanoplastics, at least nanopolystyrene, can bind metals such as silver. The formed complexes can modulate the uptake of silver nanoparticles and slightly modify some toxic effects of silver compounds, such as the ability to induce genotoxic and oxidative DNA damage. Ideally, these results should be confirmed using other different cell lines, due to the reported evidence showing significant differences according to the used cell line [15]. Additionally, extending the exposure times beyond 24 h should also be explored, although the effects of the exposure-time on the PSNPs uptake was only evident at low concentrations [15].

## Figures and Tables

**Table 1:** PS nanomaterials and AgNPs characterization by TEM (median size) and Zetasizer Nano ZS (mean ± SD).

	PSNPs	y-PSNPs	AgNPs
Median size (nm) (TEM)	45.91	42.42	4.48
Size (nm) (DLS)	86.33 ± 10.20	112.87 ± 3.11	137.30 ± 0.25
PdI (DLS)	0.10 ± 0.09	0.35 ± 0.02	0.76 ± 0.01
Z-potential (mV) (DLV)	−36.00 ± 7.88	−45.97 ± 3.84	−16.80 ± 0.76
Mobility (µm cm/Vs) (DLV)	−2.29 ± 0.10	−3.76 ± 0.38	−1.32 ± 0.06

## Data Availability

Not applicable.
